# Hypertension Prevalence, Awareness, Treatment, and Control and Sodium Intake in Shandong Province, China: Baseline Results From Shandong–Ministry of Health Action on Salt Reduction and Hypertension (SMASH), 2011

**DOI:** 10.5888/pcd11.130423

**Published:** 2014-05-22

**Authors:** Zhenqiang Bi, Xiaofeng Liang, Aiqiang Xu, Linghong Wang, Xiaoming Shi, Wenhua Zhao, Jixiang Ma, Xiaolei Guo, Xiaofei Zhang, Jiyu Zhang, Jie Ren, Liuxia Yan, Zilong Lu, Huicheng Wang, Junli Tang, Xiaoning Cai, Jing Dong, Juan Zhang, Jie Chu, Michael Engelgau, Quanhe Yang, Yuling Hong, Yu Wang

**Affiliations:** Author Affiliations: Zhenqiang Bi, Aiqiang Xu, Xiaolein Guo, Jiyu Zhang, Jie Ren, Zilong Lu, Junli Tang, Jing Dong, Jie Chu, Academy of Preventive Medicine, Shandong University, Jinan, China, and Shandong Center for Disease Control and Prevention, Jinan, China; Xiaofeng Liang, Xiaoming Shi, Wenhua Zhao, Huicheng Wang, Juan Zhang, Chinese Center for Disease Control and Prevention, Beijing, China; Linghong Wang, Jixiang Ma, Liuxia Yan, Xiaoning Cai, National Center for Chronic and Noncommunicable Disease Control and Prevention, Chinese Center for Disease Control and Prevention, Beijing, China; Xiaofei Zhang, Second Affiliated Hospital of Zhejiang University College of Medicine, Hangzhou, China; Michael Engelgau, Quanhe Yang, Yuling Hong, Centers for Disease Control and Prevention, Atlanta, Georgia.

## Abstract

**Introduction:**

In China, population-based blood pressure levels and prevalence of hypertension are increasing. Meanwhile, sodium intake, a major risk factor for hypertension, is high. In 2011, to develop intervention priorities for a salt reduction and hypertension control project in Shandong Province (population 96 million), a cross-sectional survey was conducted to collect information on sodium intake and hypertension prevalence, awareness, treatment, and control.

**Methods:**

Complex, multistage sampling methods were used to select a provincial-representative adult sample. Blood pressure was measured and a survey conducted among all participants; condiments were weighed in the household, a 24-hour dietary recall was conducted, and urine was collected. Hypertension was determined by blood pressure measured on a single occasion and self-reported use of antihypertension medications.

**Results:**

Overall, 23.4% (95% confidence interval [CI], 20.9%–26.0%) of adults in Shandong were estimated to have hypertension. Among those classified as having hypertension, approximately one-third (34.5%) reported having hypertension, approximately one-fourth (27.5%) reported taking medications, and one-seventh (14.9%) had their blood pressure controlled (<140/<90 mm Hg). Estimated total average daily dietary sodium intake was 5,745 mg (95% CI, 5,428 mg–6,063 mg). Most dietary sodium (80.8%) came from salt and high-salt condiments added during cooking: a sodium intake of 4,640 mg (95% CI, 4,360 mg–4,920 mg). The average daily urinary sodium excretion was 5,398 mg (95% CI, 5,112 mg–5,683 mg).

**Conclusion:**

Hypertension and excessive sodium intake in adults are major public health problems in Shandong Province, China.

## Introduction

Population-based blood pressure levels and the prevalence of hypertension estimated by using surveillance data have increased rapidly in the past 4 decades in China; the prevalence of hypertension increased from 5% in 1959 to 18% in 2002 (1,2). Dietary salt (sodium chloride) intake is high in China (3,4). Excess dietary sodium intake can cause hypertension, a leading risk factor for cardiovascular disease, which now accounts for approximately 40% of all deaths in China (5). In 2002, the China Health and Nutrition Survey found that approximately 80% of Chinese adults consumed more than the daily salt limit of 6 g recommended by the Chinese Nutrition Society (1). Reducing population-level dietary sodium intake could substantially reduce blood pressure levels and decrease risk for cardiovascular disease in China (6,7).

Shandong Province is the second most populous province (96 million) in China and is the birthplace of Shandong cuisine, famous for containing more salt and soy sauce than other Chinese cuisines. In 2002 in Shandong Province, daily intake of salt was 12.6 g, and 25% of those older than 15 years had hypertension (Z.B. et al, unpublished data). To address the prevention and control of hypertension and the reduction of dietary sodium consumption, the Chinese Ministry of Health and the Shandong government in 2011 collaboratively launched the Shandong–Ministry of Health Action on Salt Reduction and Hypertension (SMASH), 2011–2015. The goal of SMASH is to reduce daily salt intake to 10 g/d by 2015 and increase awareness and control of hypertension among adults in Shandong.

Since the 2002 China Health and Nutrition Survey, no provincial-representative study of salt intake and hypertension prevalence in Shandong has been conducted. The major objectives of the SMASH baseline survey were to assess the level and source of salt intake and to characterize the prevalence of hypertension in Shandong Province. The findings from this survey are being used to develop and target priority interventions to prevent hypertension, improve its control, and lower sodium intake in the Shandong population.

## Methods

### Sample size and sampling frame

Conducted during June and July 2011, the SMASH baseline survey was a population-based cross-sectional survey of residents aged 18 to 69 years. The required sample size to estimate population prevalence of hypertension was 15,153. For salt intake estimation, the required sample size was 2,088.

**Figure 1:**
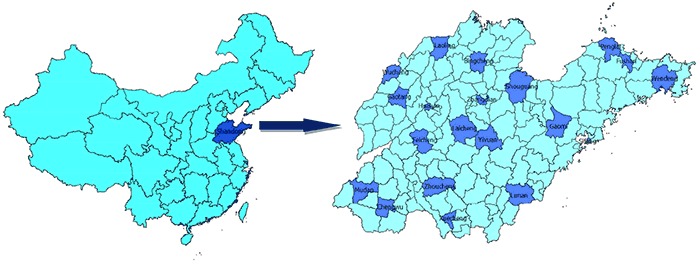
Location of the sampled countries/districts in Shandong Province, China, Shandong–Ministry of Health Action on Salt Reduction and Hypertension baseline survey, 2011.

Participants aged 18 to 69 years living in Shandong Province, who were without disability and mental disorders, were eligible to participate in the survey. We used complex, 4-stage cluster sampling to select the participants. First, we selected 20 counties/districts from 140 counties and districts after stratification by geographic distribution and by residence status (Figure 1). Second, using proportional probability sampling, we selected 3 townships (in rural areas) or 2 streets (in urban areas) from each selected county/district. Then, also using proportional probability sampling, we selected 3 villages (in rural areas) or neighborhood communities (in urban areas) from each sampled township (rural) or street (urban). Finally, in each selected village and neighborhood community, we randomly selected 100 adults from the local residents list. A total of 15,600 participants aged 18 to 69 years were selected from 13,010 households among 156 communities and villages.

From this larger sample, we also randomly selected a subsample for additional measures. Among each previously selected township and street, we randomly selected 1 village and 1 neighborhood community. Then, we randomly selected 42 participants from the larger sample in each selected village and neighborhood community. Overall 2,184 adult participants were selected for this subsample.

### Measurements

All sampled individuals were invited to participate in the survey (Appendix) and physical examination. A close-ended questionnaire was administered face-to-face by trained public health staff. We collected information on individual sociodemographics, self-reported history of hypertension and diabetes, as well as the lifestyle habits of smoking, alcohol use, physical activity, and diet. We also collected data on their knowledge of the health outcomes of sodium intake and hypertension, perceptions of salt consumption, and attitudes and intentions toward reducing salt intake.

Height, weight, waist circumference, and blood pressure were measured by trained health staff using standardized methods (8). Blood pressure was measured 3 times every 5 minutes during a single occasion by electronic sphygmomanometer (HEM-7071, Omron Corporation, Kyoto, Japan) while participants were in the sitting position.

Among the subsample, a 24-hour dietary recall, which included a weighing of condiments in the household, was administered, and a 24-hour urine sample was collected. (At the same time the urine was collected, a fasting blood sample was also collected, but the results of these blood samples will be published elsewhere.) The 24-hour dietary recall took place during 3 consecutive days, usually Thursday, Friday, and Saturday (9). Trained health staff visited each selected household and weighed all condiments (ie, salt, low-sodium salt, soy sauce, monosodium glutamate, vinegar, chicken essential, bean sauce, sweet flour sauce, chili sauce, and shrimp sauce) directly at the start of the first 24-hour dietary recall and at the end of the last 24-hour dietary recall. Each selected household was asked to record the purchase or spillage (or other wastage) of any condiments during the survey days and report these to the health staff during their final visit. Individuals were trained to record their entire food diary every day into a standardized table and report the portion of each item consumed. The total individual dietary sodium intake was the combination of sodium estimated from condiments and the dietary recall. We used the sodium reference values from China Food Composition Table 2004 (10) to calculate the sodium content for all condiments and foods.

Participants were instructed on the collection of a standard 24-hour urine sample and then given a standardized urine-collection container (11). Trained health staff controlled the quality of urine sample collection at each survey field site. They recorded both the beginning time and ending time of urine collection for each participant and used standard questionnaires to evaluate the completeness of the 24-hour urine collection. Urinary sodium, potassium, creatinine, and microproteinurina were then assessed by using standard laboratory assays. Urinary sodium and potassium were examined by the ion-selective electrode method (12).

### Data management and analysis

Data were double-entered in EpiData Entry 3.1 (The EpiData Association, Odense, Denmark); cleaning and archiving was done by using established protocols. Extreme and missing data were manually checked to the original paper records, and data entry and logical errors were corrected or updated.

Hypertension was defined according to the 2010 Chinese Guidelines for the Management of Hypertension (13). Participants were classified as hypertensive if their mean systolic blood pressure (SBP) was 140 mm Hg or greater or if their mean diastolic blood pressure (DBP) was 90 mm Hg or greater or if they self-reported currently taking antihypertension medication in the previous 2 weeks (13). Awareness of hypertension was defined as self-report of any previous diagnosis of hypertension by a health care professional. Treatment of hypertension was defined as self-reported use of antihypertension medication. Control of hypertension was defined as treatment of hypertension associated with a mean SBP of less than 140 mm Hg and DBP of less than 90 mm Hg (13).

Sample weights were developed to represent the entire population in Shandong Province. The total weight was determined by the design weight and poststratification weight (14). The design weight was calculated by accounting for cluster design, strata, and individual. The general population distribution of Shandong Province in 2009 was used for poststratification weight calculations. Taylor-series linearization was used to estimate the variance of mean and proportion and to calculate 95% confidence intervals (CIs). Two sets of weights were calculated for the overall sample and for the subsample.

We calculated the weighted SBP and DBP, the prevalence of hypertension, and the percentage of hypertensive participants who were aware of, taking medication for, and controlling their hypertension. We also estimated the weighted mean of dietary sodium intake and 24-hour urinary sodium excretion and the difference between the 2 measures. A *P* value less than .05 was considered significant. Statistical analyses were performed with SAS software version 9.3 (SAS Institute Inc, Cary, North Carolina).

### Ethics considerations

The survey received ethical approval from the ethics committee of the Shandong Center for Disease Control and Prevention. All participants signed informed consent before participating in the survey.

## Results

**Figure 2:**
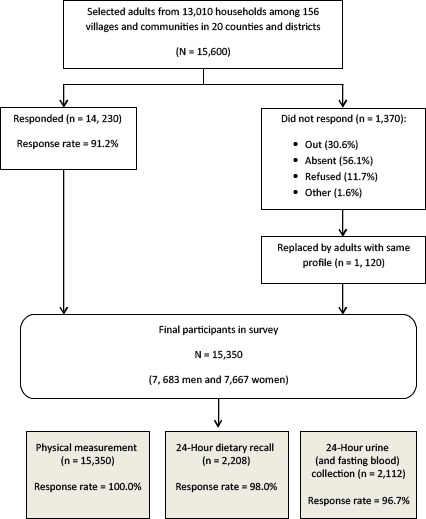
Participant flow in the Shandong–Ministry of Health Action on Salt Reduction and Hypertension baseline survey in Shandong Province, China, 2011. In the list of reasons for nonresponse, “out” is defined as not residing in hometown for an extended period because of working or education purposes; “absent” is defined as not at home on the day the survey was conducted.

Among 15,600 individuals selected, 14,230 responded and participated in the survey (response rate, 91%). Of the 1,370 nonresponders, 1,120 were replaced by adults with similar profiles from the same community or village. A total of 15,350 participated in the survey; a subsample of 2,208 participated in the 24-hour dietary recall, and 2,112 participated in the 24-hour urine collection (Figure 2).

Among all participants, 50% were men, 50% were women, 31% lived in urban areas, and 69% lived in rural areas. The weighted mean age was 40.7 years (95% CI, 40.2 y–41.2 y). Almost all participants (99.4%) were Han; most (92.8%) had less than a high school education (Table 1). Overall, the weighted mean (standard deviation) was 121 (18.3) mm Hg for SBP and 79 (11.7) mm Hg for DBP. Men had higher mean SBP and DBP than women (Table 2).

**Table 1:** Characteristics of Study Participants (N = 15,350) in Shandong Province, China, SMASH Baseline Survey, 2011

Characteristic	Total		Urban		Rural	
	n	% (95% CI)^a^	n	% (95% CI)^a^	n	% (95% CI)^a^
**Sex**						
Male	7,683	50.4 (49.7–51.1)	2,391	50.1 (48.9–51.3)	5,292	50.6 (49.6–51.6)
Female	7,667	49.6 (48.9–50.3)	2,413	49.9 (48.7–51.1)	5,254	49.4 (48.4–50.4)
**Ethnicity**						
Han	15,262	99.4 (99.2–99.6)	4,769	99.3 (98.9–99.7)	10,493	99.5 (99.3–99.7)
Other^b^	88	0.6 (0.4–0.8)	35	0.7 (0.3–1.1)	53	0.5 (0.3–0.7)
**Education, y**						
<9	11,782	77.3 (73.0–81.5)	2,841	62.6 (45.5–79.7)	8,941	83.5 (80.7–86.2)
9–11	2,372	15.5 (13.3–17.6)	1,099	21.6 (13.6–29.6)	1,273	12.9 (11.0–14.7)
≥12	1,196	7.3 (4.8–9.7)	864	15.8 (5.9–25.7)	332	3.6 (2.4–4.9)
**Smoking status^c^, by sex**						
**Men**						
Never	3,172	40.3 (36.3–44.3)	1,099	39.5 (29.0–50.0)	2,163	40.6 (36.1–45.1)
Former	637	8.3 (7.2–9.3)	216	9.2 (6.1–12.4)	421	7.9 (6.9–8.9)
Current	3,874	51.4 (48.0–54.9)	1,166	51.2 (43.7–58.7)	2,708	51.5 (47.1–55.9)
**Women**						
Never	7,417	96.8 (94.9–98.8)	2,351	98.1 (95.9–100)	5,066	96.3 (93.5–99.0)
Former	63	0.8 (0.3–1.3)	12	0.4 (0-1)	51	1.0 (0.3–1.6)
Current	187	2.4 (0.9–3.9)	50	1.6 (0-3.1)	137	2.7 (0.6–4.8)
**BMI^d^**						
Low weight	664	4.5 (4.1–5.0)	211	4.7 (3.9–5.5)	453	4.4 (3.8–5.1)
Normal	6,930	46.1 (43.9–48.3)	2,023	43.4 (37.8–49.0)	4,907	47.2 (44.6–49.8)
Overweight	5,081	32.8 (31.5–34.1)	1,642	33.2 (29.9–36.5)	3,439	32.6 (31.0–34.2)
Obese	2,662	16.6 (14.9–18.3)	922	18.6 (15.2–22.1)	1,740	15.7 (13.7–17.8)

Abbreviations: SMASH, Shandong–Ministry of Health Action on Salt Reduction and Hypertension; CI, confidence interval; BMI, body mass index.

a Percentages were weighted to represent the total population of Shandong adults aged 18 to 69 years poststratified by age and sex.

b Other ethnicities were Zhuang, Man, Hui, Miao, Uyghur, Yi, Tujia, Mongol, Korean, and Tibetan.

c Never was defined as those who had smoked fewer than 100 cigarettes in a lifetime. Those who had smoked 100 or more than 100 cigarettes in a lifetime were defined as smoker; of smokers, those who were currently smoking were classified as current and those who had quit were classified as former.

d Calculated from measured height and weight. Only participants for whom height and weight data were available (n = 15,337) were included in analysis. Data on 13 people (6 urban, 7 rural) were missing. BMI was calculated as weight in kilograms (kg) divided by height in meters squared (m^2^). BMI < 18.5 classified as low weight; 18.5 to <24.0 as normal; 24.0 to <28.0 as overweight; and ≥28.0 as obese, according to Chinese overweight and obesity guidelines (15).

**Table 2:** Mean Systolic and Diastolic Blood Pressure and Hypertension Prevalence, Awareness, and Control Among Adults in Shandong Province, China (N = 15,350), SMASH Baseline Survey, 2011^a^

Measure	Total, Mean (95% CI)^b^	Urban, Mean (95% CI)^b^	Rural, Mean (95% CI)^b^
**Systolic blood pressure, mm Hg^c^**			
Men	124.2 (122.9–125.6)	122.5 (120.0–125.1)	124.9 (123.3–126.6)
Women	117.9 (116.4–119.3)	114.6 (112.2–117.0)	119.3 (117.5–121.0)
Total	121.1 (119.7–122.4)	118.6 (116.4–120.8)	122.1 (120.4–123.8)
**Diastolic blood pressure, mm Hg^c^**			
Men	80.4 (79.3–81.5)	79.8 (77.4–82.2)	80.6 (79.2–82.0)
Women	77.3 (76.4–78.2)	76.0 (74.2–77.7)	77.9 (76.7–79.1)
Total	78.9 (77.9–79.9)	77.9 (75.9–79.8)	79.3 (78.0–80.5)
**Prevalence of hypertension^d^**			
Men	25.7 (22.9–28.6)	24.1 (18.0–30.1)	26.4 (22.8–30.0)
Women	21.1 (18.6–23.7)	17.5 (13.4–21.6)	22.7 (19.4–26.0)
Total	23.4 (20.9–26.0)	20.8 (16.0–25.6)	24.6 (21.3–27.8)
**Awareness of hypertension^e^^, ^ f**			
Men	31.5 (27.2–35.9)	41.6 (35.4–47.7)	27.7 (22.1–33.3)
Women	38.1 (32.5–43.6)	44.8 (36.3–53.2)	35.9 (28.5–43.2)
Total	34.5 (29.8–39.2)	42.9 (37.2–48.7)	31.4 (25.1–37.7)
**Treatment of hypertension^e^^, ^ g**			
Men	24.1 (20.5–27.7)	32.6 (26.3–39.0)	20.8 (16.5–25.1)
Women	31.7 (27.0–36.4)	39.7 (28.7–50.7)	29.1 (23.4–34.8)
Total	27.5 (23.6–31.4)	35.6 (28.2–43.0)	24.6 (19.8–29.4)
**Control of hypertension^e^^, ^ h**			
Men	13.7 (11.6–15.8)	16.1 (12.5–19.7)	12.8 (10.1–15.5)
Women	16.4 (14.1–18.6)	19.9 (11.9–27.8)	15.2 (13.2–17.3)
Total	14.9 (13.0–16.8)	17.7 (12.6–22.7)	13.9 (11.7–16.1)

Abbreviations: SMASH, Shandong–Ministry of Health Action on Salt Reduction and Hypertension; CI, confidence interval.

a Values are percentage (95% CI), unless otherwise indicated.

b Means and percentages were weighted to represent the total population of Shandong adults aged 18 to 69 years poststratified by age and sex.

c The average of 3 blood pressure measurements on a single occasion.

d Hypertension was determined by blood pressure measured on a single occasion and self-reported use of antihypertension medications. Participants were designated as having hypertension if mean systolic blood pressure was ≥140 mm Hg or diastolic blood pressure was ≥90 mm Hg or if they self-reported currently taking antihypertension medication in the previous 2 weeks. This definition differs from the definition applicable in clinical settings, which requires readings averaged during 2 or more occasions.

e Calculated among participants classified as having hypertension as defined in the previous footnote.

f Awareness of hypertension was defined as self-report of any previous diagnosis of hypertension by a health care professional.

g Treatment of hypertension was defined as self-reported use of antihypertension medication.

h Control of hypertension was defined as treatment of hypertension associated with a mean systolic blood pressure of less than 140 mm Hg and diastolic blood pressure of less than 90 mm Hg (13).

The weighted prevalence of hypertension was 23.4% (95% CI, 20.9%–26.0%). Among those classified as having hypertension, only one-third (34.5%; 95% CI, 29.8%–39.2%) were aware of their condition; about one-fourth (27.5%; 95% CI, 23.6%–31.4%) reported taking antihypertension medications, and one-seventh (14.9%; 95% CI, 13.0%–16.8%) had their blood pressure controlled (Table 2). The prevalence of hypertension was not different between rural and urban areas (*P* = .12). Among those with hypertension, awareness (*P* = .001) and treatment (*P* = .002) of hypertension were significantly lower in rural areas than in urban areas. However, we found no difference in hypertension control between rural and urban areas (*P* = .07).

**Figure 3:**
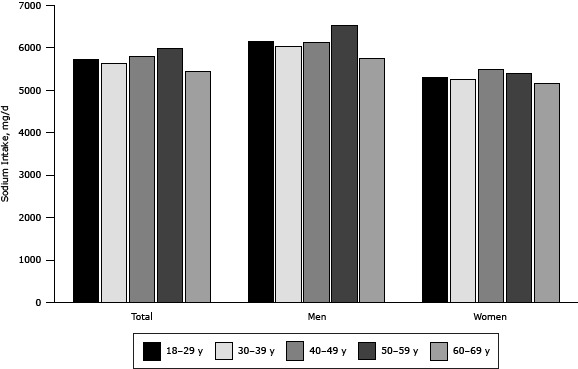
Age- and sex-specific daily dietary sodium intake of adults participating in the Shandong–Ministry of Health Action on Salt Reduction and Hypertension baseline survey in Shandong Province, China, 2011. **Table:** 

The total average daily dietary sodium intake estimated by 24-hour dietary recall was 5,745 mg (95% CI, 5,428 mg–6,063 mg) (Table 3). Rural participants consumed greater amounts of sodium than urban participants (*P* = .03), and men consumed greater amounts of sodium than women (*P*< .001). Dietary sodium intake increased by age among participants younger than 60 years, peaked in the group aged 50 to 59, and then decreased (Figure 3). The trend of sodium intake across age groups was similar among men and women.

**Table 3:** Differences in Daily Dietary Sodium Intake and Urinary Sodium Excretion Among Adults in Shandong Province, China, SMASH Baseline Survey, 2011^a^

Measure	Total	Residence		Sex	
		Urban	Rural	Male	Female
**Daily dietary sodium intake^b^^, ^ c (n = 2, 140)**					
Total	5,745 (5,428–6,063)	5,342 (5,007–5,676)^d^	5,910 (5,449–6,371)^d^	6,147 (5,824–6,471)^e^	5,339 (5,006–5,673)^e^
Condiments added at cooking	4,640 (4,360–4,920)	4,236 (3,986–4,487)	4,805 (4,398–5,213)	4,861 (4,564–5,159)	4,417 (4,144–4,690)
Salt added at cooking	3,638 (3,397–3,878)	3,376 (2,852–3,899)	3,745 (3,429–4,060)	3,790 (3,540–4,041)	3,484 (3,246–3,721)
Other condiments added at cooking	1,003 (808–1,197)	861 (439–1,282)	1,061 (822–1,299)	1,071 (870–1,272)	934 (738–1,129)
Processed food	582 (498–666)	550 (412–689)	595 (480–710)	674 (573–776)	489 (409–568)
Other	523 (458–588)	555 (438–671)	510 (425–595)	611 (528–695)	433 (380–487)
**Urinary sodium excretion (n = 2,024)^b^**					
Total, mg	5,398 (5,112–5,683)	5,352 (4,379–6,327)	5,419 (5,014–5,824)	5,598 (5,269–5,925)^e^	5,184 (4,904–5,465)^e^
**Difference between dietary sodium intake and urinary sodium excretion (n = 1,914)^b^^, ^ f**					
Total, mg	352 (−30 to 733)	−47 (−716 to 622)	528 (30 to 1,025)	551 (173 to 929)	140 (−294 to 574)

Abbreviations: CI, confidence interval; SMASH, Shandong–Ministry of Health Action on Salt Reduction and Hypertension.

a All values are mean (95% CI), mg/d, unless otherwise indicated. Means were weighted to represent the total population of Shandong adults aged 18 to 69 years poststratified by age and sex.

b Salt and sodium are converted by the following equation: 1 g salt = 390 mg sodium; 1 mmol sodium = 23 mg sodium.

c The total sources of dietary sodium included 3 broad categories: condiments added at cooking, sodium from processed food, and other sources (unclassified). The broad category “condiments added at cooking” includes 2 subcategories: “salt added at cooking” and “other condiments added at cooking.”

d Difference between rural and urban residents, *P* < .05.

e Difference between male and the female residents, *P* < .05.

f Only participants with records of both 24-h dietary recall and 24-h urine collection were included in analysis.

Age group, y	Sodium Intake, mg/d		
	Total	Men	Women
18–29	5,737	6,159	5,301
30–39	5,633	6,023	5,247
40–49	5,801	6,118	5,488
50–59	5,980	6,532	5,408
60–69	5,454	5,747	5,167

Overall, most dietary sodium (80.8%; 95% CI, 79.9%–81.6%) came from condiments, including salt, added during the cooking process. The mean daily sodium intake from condiments added at cooking was 4,640 mg (95% CI, 4,360 mg–4,920 mg). Among condiments added at cooking, salt was the leading source (78.4%; 95% CI, 74.7%–82.1%) of sodium. The daily average sodium consumption from processed food was 582 mg (95% CI, 498 mg–666 mg) and accounted for 10.1% (95% CI, 9.0%–11.3%) of the total sodium intake (Table 3).

The mean 24-hour urinary sodium excretion was 5,398 mg (95% CI, 5,112 mg–5,683 mg) (Table 3). We found no difference between rural and urban residents in 24-hour urinary sodium excretion (*P* = .08); men excreted more sodium than women (*P* = .002) (Table 3). Overall, the sodium intake estimated by dietary recall was significantly correlated with urinary sodium excretion (*r* = 0.16; *P*< .001); the mean difference was 352 mg (95% CI, −30 mg to 733 mg) (Table 3).

## Discussion

Hypertension has become a nationwide epidemic in China (1,2,16). Our study found that 1 in 4 adults in Shandong Province had hypertension in 2011 — an estimated 16 million people. Other surveys, using different methods of sampling and analysis, found that hypertension is becoming common in Shandong Province (17,18). The prevalence of hypertension in Shandong in our study was lower than the pooled prevalence in the northern region (25.8%) and higher than that in the southern region of China (20.4%) (19).

We found that hypertension was more common in rural areas than in urban areas of Shandong. A study of the rural population in Shandong suggested that the prevalence of hypertension increased by 50% from 1991 to 2007 (18). A corresponding trend analysis for urban areas is not available for comparison. At the national level, hypertension among rural populations has also increased faster than that among urban populations. In 1959, the national prevalence of hypertension among urban residents was 1.5 times the prevalence among rural residents (2). By 2002, the urban–rural disparity was much diminished (urban-to-rural ratio = 1.2) (16), and by 2010, the disparity had further diminished (urban-to-rural ratio = 1.05) (20). Therefore, it is not surprising that the burden of hypertension is larger in the rural than the urban population in Shandong now. Economic development in China has led to greater availability of blood pressure measurements than before in rural areas; such availability has allowed the detection and diagnosis of hypertension among those with previously undetected hypertension. In addition, the prevalence of obesity, a major risk factor for hypertension, increased faster in rural areas than in urban areas (21).

Hypertension control in Shandong Province improved since the 2002 national survey (15% in 2011 vs 5% in 2002) (16). However, hypertension control was much lower than that found in the United States and other countries (22,23). The low awareness of blood pressure levels is a crucial factor in hypertension control. Campaigns to increase blood pressure screening and detection should be emphasized in SMASH interventions. In addition, our study showed that among those aware of their hypertension, less than half (43.2%) had their blood pressure controlled. The lower control rate might be related to poor adherence to medication use (24). Health education on adherence to using antihypertension medication should be promoted by primary health service centers in the SMASH program. In addition, salt substitute, a salt with reduced sodium and increased potassium, is now being used in the China Rural Health Initiative in 5 northern Chinese provinces; if proved effective, using salt substitute might be a strategy to reduce sodium consumption and control hypertension (25).

We found dietary salt intake among Shandong adults was high and changed little during the past 10 years. Reducing the population levels of salt intake is challenging. One major barrier is changing the salty taste preference and appetite for current salt content typified by traditional Shandong cuisine. Lowering salt content in foods may lead to loss of palatability and may be unacceptable for individual taste preferences (26). However, gradual sodium reduction (10%–15% over multiple years) can be implemented and might lead to adjustment of the salt taste. This strategy was adopted by salt reduction programs in England and other countries (27,28). The Food Standards Agency in England is working with the food industry progressively toward the 6-g-per-day salt intake target. We have adopted the same strategy in SMASH and plan to achieve salt-reduction goals by moderately and gradually reducing levels of salt from high-sodium food.

Condiments added during food preparation are the major source of sodium intake; processed foods accounted for the minority of sodium intake in Shandong. This finding is consistent with the findings of other studies in China (1,3,4), and it suggests that salt reduction in food preparation in households, restaurants, and canteens should be set as high priority for SMASH interventions and that household food preparers and restaurant chefs should be the key target audience for the message.

Sodium consumption can be measured by dietary sodium intake recalls and 24-hour urinary sodium excretion measurement. However, methodological challenges limit the accurate measurement of sodium intake in both methods (29). Dietary surveys are inaccurate when respondents do not report their food intake accurately. In addition, accounting for the waste and spillage of condiments is challenging, particularly in a field study that includes a large sample of households. Twenty-four-hour urine collection misses approximately 10% of sodium intake excreted in sweat and feces. Validation of urine collection completeness is also difficult. The p-aminobenzoic acid (PABA) test is recommended for 24-hour urine collection, but the utility and the safety of PABA tests for population surveys have not been established (11). The alternative biomarker creatinine has moderate sensitivity in detecting urine incompleteness (30).

Our study has many strengths. First, it was conducted in a large representative sample of the general population in Shandong Province. Second, we used a combination of methods to estimate sodium intake: 24-hour dietary recall, weighing of condiments, and 24-hour urine collection. Third, we had a strict quality-control process in all phases of the study to ensure that data were reliable.

Our study also has several limitations. First, we used the surveillance definition of hypertension, and we took blood pressure measurements on a single occasion (although 3 measurements were taken on that 1 occasion). Blood pressure varies over time in individuals, and 2 or more blood pressure readings on 2 or more occasions are required for a clinical diagnosis of hypertension (31). Second, data on wastage of condiments were collected by self-report, which may have led to an overestimation or underestimation of the sodium intake derived from this source. Third, we didn’t use PABA as the biomarker to validate 24-hour urine completeness. We used creatinine as an alternative biomarker; doing so may have resulted in the inclusion of noneligible samples or the exclusion of eligible samples. Fourth, approximately 7% of the nonresponding participants were substituted by nonrandomly selected individuals. However, after we removed those substituted participants and repeated all analyses, we found similar results, which suggest that the substitution had little effect on our study results.

In conclusion, we found significant levels of hypertension in Shandong Province, particularly in rural areas. Control of hypertension has improved but is very low. Sodium intake is high, and condiments added during home food preparation accounts for most of the sodium intake. Opportunities have been identified for strategic efforts in targeting hypertension prevention and control and reducing sodium intake.

## Appendix. Text of Questionnaire on Salt and Hypertension-Associated Knowledge, Attitude, and Practice in Shandong–Ministry of Health Action on Salt Reduction and Hypertension (SMASH) Project Baseline Survey Among Adults in Shandong, China, 2011

Question 1: Do you know the diagnosis criteria of hypertension?

1. ≥140/90 mm Hg
2. ≥130/80 mm Hg
3. ≥120/80 mm Hg
4. Don’t know

Question 2: Do you know the consequences caused by hypertension [multiple choices]?

1. Stroke
2. Coronary heart disease
3. Kidney disease
4. Eye disease
5. Don’t know

Question 3: Do you know the risk factors that could lead to hypertension?

1. Overweight or obese
2. Habitual excessive alcohol drink
3. Habitual high-sodium diet
4. Hypertension family history
5. Hyperglycaemia and hyperlipidemia
6. Aging
7. Mental stress
8. Don’t know

Question 4: Do you think any of the following statements are correct [multiple choices]?

1. It would be out of strength if we intake less salt
2. Too much salt could increase blood pressure
3. Whenever we are healthy, we do not need to reduce salt intake
4. Too much salt could lead to osteoporosis
5. Don’t know

Question 5: Do you know the daily salt intake recommendation in China?

1. <2 g
2. <6 g
3. <9 g
4. <12 g
5. Don’t know

Question 6: Do you know that reducing salt intake could decrease blood pressure?

1. Yes, I know
2. No, I don’t know

Question 7: Do you know excessive salt intake could cause what disease [multiple choices]?

1. Hypertension
2. Stroke
3. Myocardial infarction
4. Kidney disease
5. Stomach cancer
6. Osteoporosis
7. Don’t know

Question 8: How do you assess your salt intake?

1. Too little
2. Moderate
3. Too much

Question 9: After you have known the unfavorable consequence of excessive salt intake, do you have intention to reduce salt intake?

1. Yes, I have salt reduction intention (Please go to Question 11)
2. No, I don’t (Please go to Question 10)
3. Don’t know (Please go to Question 11)

Question 10: Could you please tell us the reason why you don’t plan to reduce salt?

1. Unfavorable food taste of decreased salt added
2. It would be out of strength once we decrease salt intake
3. There are no adverse outcomes by taking too much salt
4. I don’t know

Question 11: Have you used a scaled salt spoon to control your salt added in cooking in your household?

1. Yes, I have (Please go to Question 12)
2. No, I haven’t (Please go to Question 16)
3. I don’t know (Please go to Question 16)

Question 12: Where did your family get the scaled salt spoon?

1. From the health service centers
2. It was a gift of purchasing other products
3. I bought it myself
4. Other, please clarify___________

Question 13: Do you know the method of using the scaled salt spoon?

1. Yes, I know
2. No, I don’t

Question 14: Could your family use the scaled salt spoon correctly?

1. Yes, we could
2. No, we couldn’t
3. Don’t know

Question 15: Which type of scaled salt spoon did your family use?

1. 2-gram-scaled salt spoon
2. 3-gram-scaled salt spoon
3. 6-gram-scaled salt spoon
4. Other, please clarify.___________

Question 16: Do you think the CDC should advocate the low-sodium diet in the mass media?

1. Yes, I agree
2. No, I don’t
3. Don’t know

Question 17: Have you received health education on the low-sodium diet?

1. Yes, I have (Please go to Question 18)
2. No, I haven’t (Please go to Question 19)
3. Don’t know (Please go to Question 19)

Question 18: We will read some health education channels to you. Please select the major 3 channels that you have used to receive health education on the low-sodium diet.

1. Brochures or folding
2. Broadcast, television
3. Newspaper, magazine
4. Column
5. Health professionals
6. Family member
7. Colleagues or friends
8. Slogans
9. Other, please clarify_____________

Question 19: Have you advocated the low-sodium diet to your friends or colleagues?

1. Yes, I have
2. No, I haven’t

Question 20: Do you think that low-sodium diet could affect food taste?

1. Yes, it will have great effect
2. Yes, it will have moderate effect, but quite acceptable
3. No, I don’t think it could affect it
4. Don’t know

Question 21: Which population do you think population should adhere to the low-sodium diet (multiple choices)?

1. Patients with hypertension
2. Patients with stroke
3. Patients with coronary heart disease
4. Don’t know

Question 22: Have you noticed food labeling when you buy processed food?

1. Yes, I have
2. No, I haven’t
3. Don’t know

Question 23: What is your attitude on listing the sodium content in the food label on the processed food?

1. Positive
2. Negative
3. Don’t know

Question 24: Do you think that food labeling on sodium content on processed foods have played a role in your choice of low-sodium food?

1. Yes, it has
2. No, it hasn’t
3. Don’t know

Question 25: What is your attitude toward the low-sodium diet?

1. Support
2. Oppose
3. Neutral

Question 26: Have you taken actions on salt reduction?

1. Yes, I have (Please go to Question 27)
2. No, I haven’t (Please go to Question 28)
3. Don’t know (Please go to Question 28)

Question 27: Which salt reduction measurements have you taken [multiple choices]?

1. Reduce added salt in cooking
2. Update salt-adding procedure in food cooking, ie, postpone salt adding once food is ready
3. Reduce high-sodium condiments use
4. Reduce the consumption of pickles (high-sodium food)
5. Choose low-sodium processed food
6. Replace the salt with condiments that were low sodium, ie, vinegar
7. Use onion, ginger, garlic to increase the taste and reduce the salt adding
8. Other, please clarify
9. Don’t know

Question 28: Have you heard of low-sodium salt (salt substitute)?

1. Yes, I have (Please go to Question 29)
2. No, I haven’t (End)

Question 29: Do you know that low-sodium salt(salt substitute) has better effects on blood pressure control than regular salt?

1. Yes, I know
2. No, I don’t

Question 30: Have you used low-sodium salt?

1. Yes, I have (End)
2. No, I haven’t (End)
